# Supplementary material for: Intrinsic factors and CD1d1 but not CD1d2 expression levels control invariant natural killer T cell subset differentiation
Source: Nat Commun. 2023 Dec 1;14:7922. doi: 10.1038/s41467-023-43424-7 (PMC10692182; doi:10.1038/s41467-023-43424-7)
Supplement: Supplementary file 1 — Supplementary Information [file 41467_2023_43424_MOESM1_ESM.pdf]

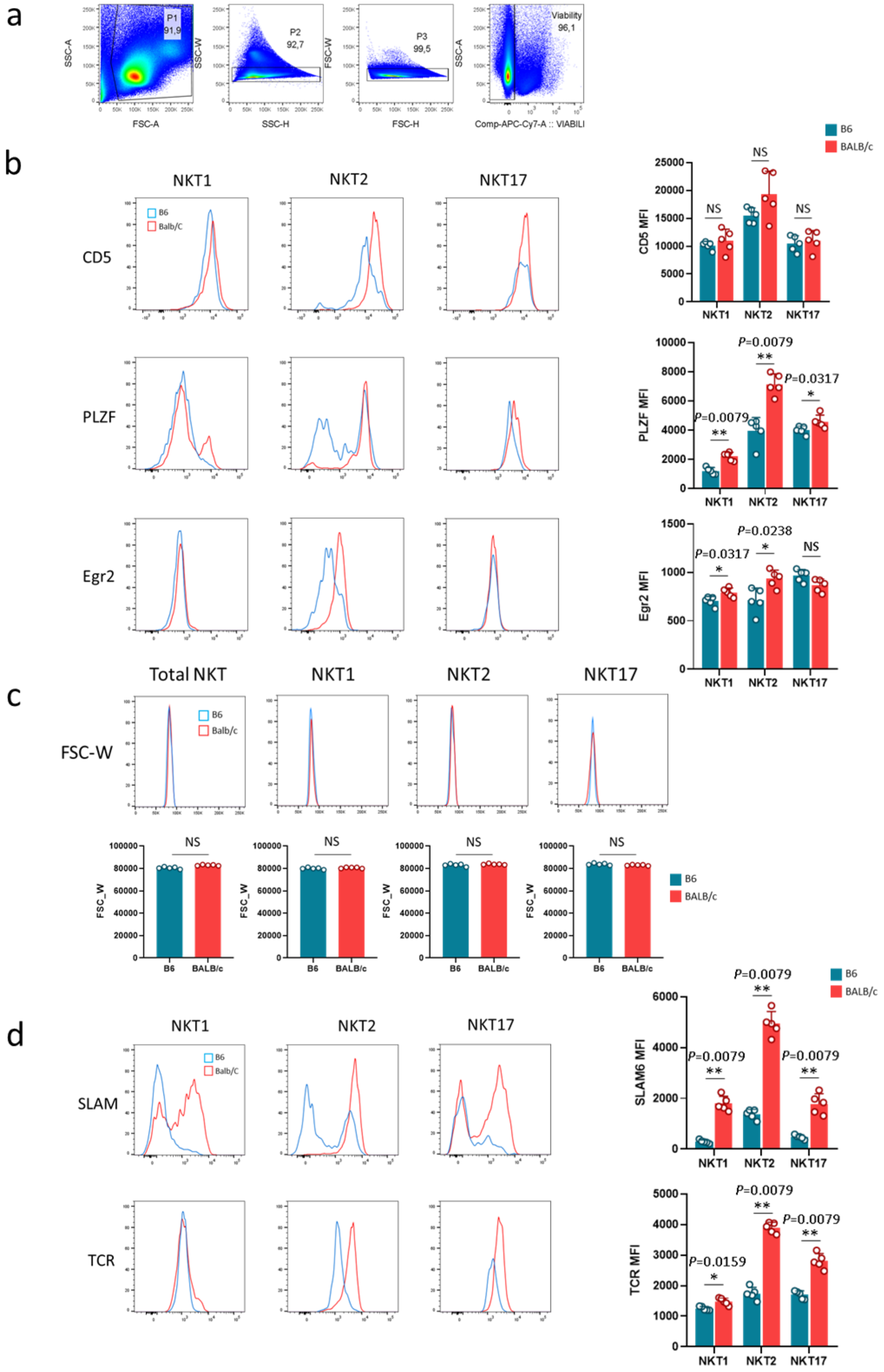

Supplementary Figure 1: **Differential TCR signal strength in B6 and BALB/c NKT cells**. **a**. Gating strategy for NKT cell analysis **b**. Representative staining for CD5, PLZF, and Egr2, in the NKT cell subsets (NKT1, NKT2, and NKT17) from B6 (n=5) and BALB/c (n=5) mice. Individual/mean + SEM of mean MFI of these markers are shown to the right of each histogram plot. **c**. Representative histogram plots showing cell size (FSC-W) in total thymic NKT and NKT cell subsets (NKT1, NKT2, and NKT17) in B6 (n=5) and BALB/c (n=5) mice. Individual/mean + SEM of mean cell size are shown in the lower panel. **d**. Representative staining for SLAM6 and TCR in thymic NKT cell subsets in B6 (n=5) and BALB/c (n=5) mice. Individual/mean + SEM of mean MFI for these markers are shown in the right panel. Data are representative of five experiments in b., and four experiments in c., and d., with 7-8-week-old mice. Statistics were calculated with the nonparametric Mann-Whitney test, two-sided. *$P < 0.05$, **$P < 0.01$. NS not significant ($P > 0.05$). Source data are provided as a Source Data file.

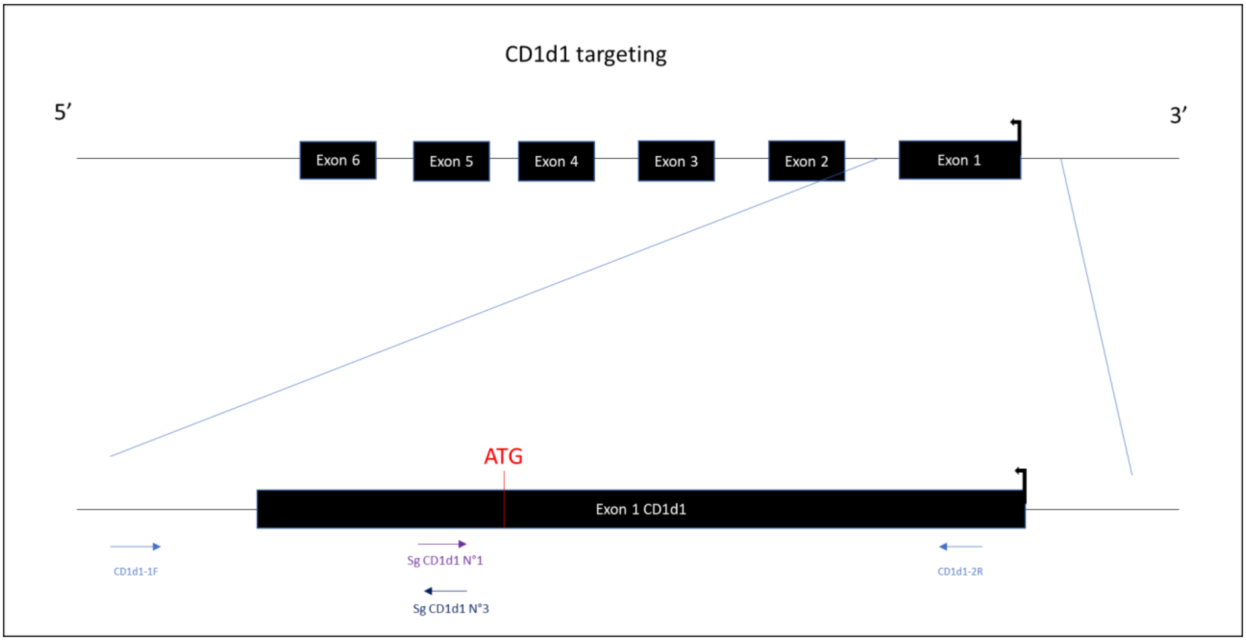

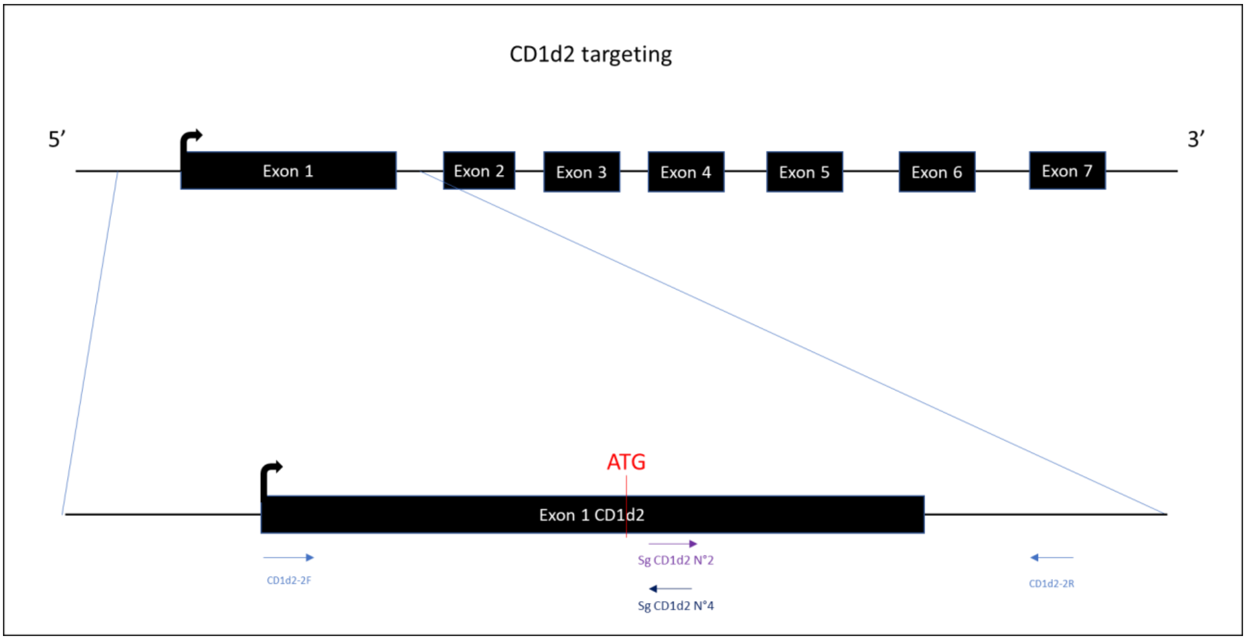

Supplementary Figure 2: Generation of CD1D1 and CD1D2 knock-out mice. CD1d isomorph knock-out BALB/c mice were generated by Crispr-Casp9 technology. For CD1d1 KO mice (named CD1d2 mice), founders had an additional T in position 17, starting ATG, which induced a downstream STOP codon producing a truncated 19-aa protein. For CD1d2 KO mice (named CD1d1 mice), a T at position 23 was deleted in founders inducing a downstream STOP codon. Thus, from the start codon ATG to the new STOP, a 38-aa protein was produced. For details, see supplemental material and methods.

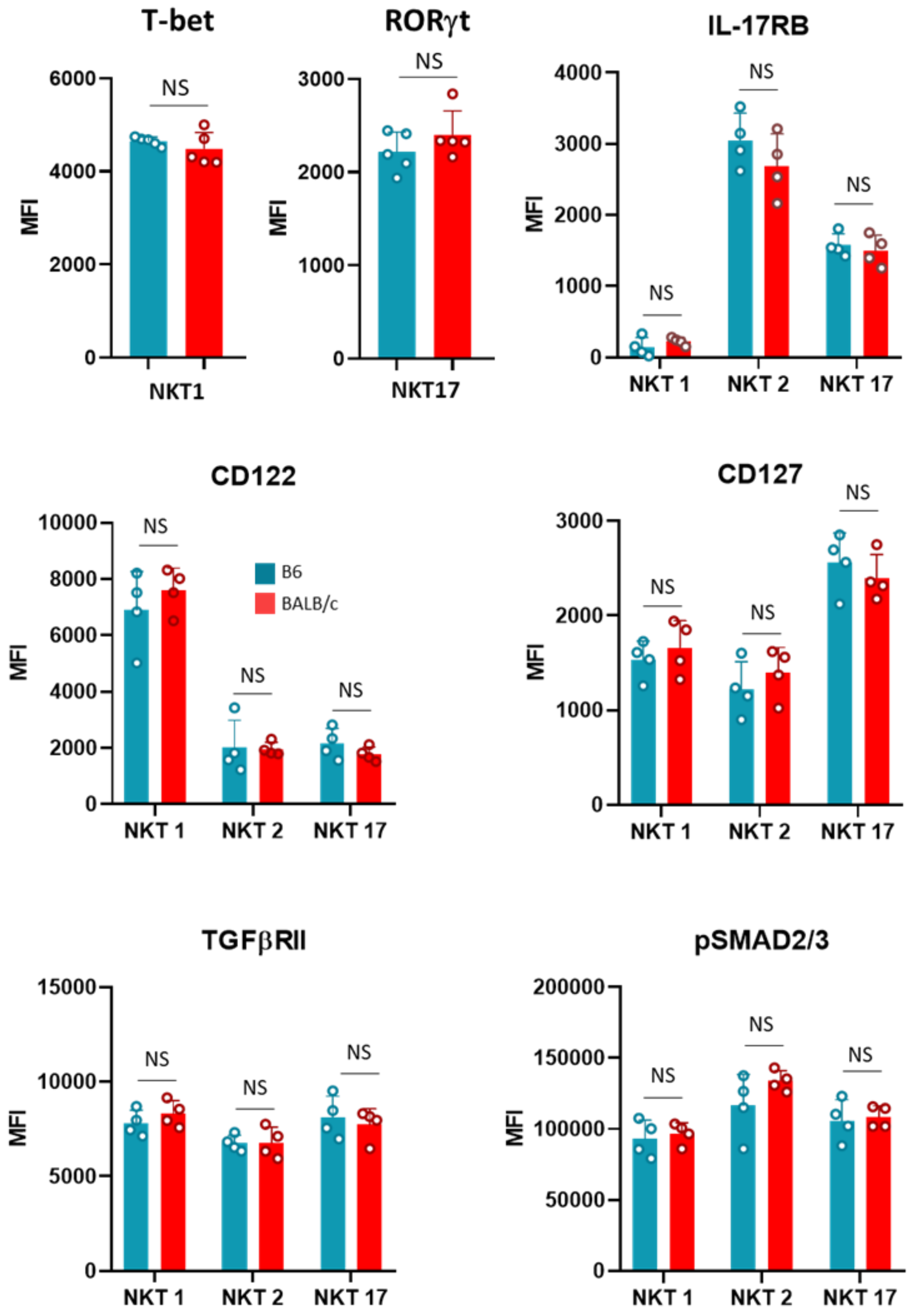

Supplementary Figure 3: Expression of intrinsic factors related to the development and/or function of NKT1 (T-bet, IL-15R (CD122)), NKT2 (IL-25R: IL-17RB), and NKT17 (RORγt, IL-7R, TGFbRII, phospho-SMAD2/3) in NKT cell subsets of B6 and BALB/c mice. Individual/ average MFI +SEM for the indicated markers in B6 (n=5 for T-bet and RORγt and 4 for the other markers) and BALB/c (n=5 for T-bet and RORγt and 4 for the other markers) mice. Data are representative of seven experiments for T-bet and RORγt and two experiments for all the other markers with 7-8-week-old mice. Statistics were calculated with the nonparametric Mann-Whitney test, two-sided, NS not significant ($P > 0.05$). Source data are provided as a Source Data file.

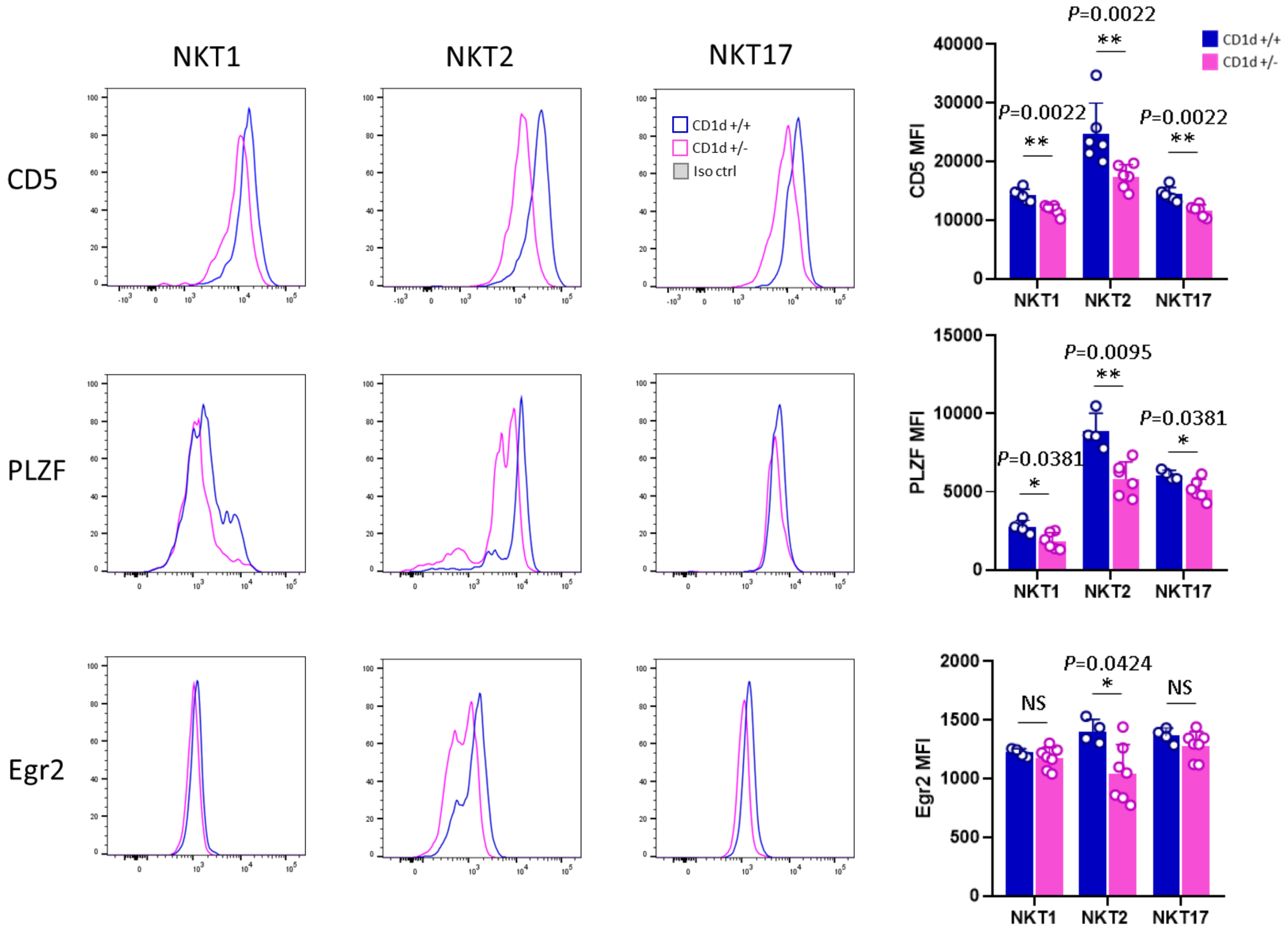

Supplementary Figure 4: **CD1d expression levels affect NKT cell subset composition and function.** Representative staining for CD5, PLZF, and Egr2, in the thymic NKT cell subsets from CD1d+/+ (n=6 for CD5, n=4 for PLZF, and n=4 for Egr2) and CD1d+/- (n=6 for CD5, n=6 for PLZF, and 7 for Egr2) mice. Individual/mean + SEM of mean frequency are shown in the right panel. Data are representative of four experiments with 7-8-week-old mice, in each experiment. Statistics were calculated with the nonparametric Mann-Whitney test, two-sided, **P* < 0.05, ***P* < 0.01. NS not significant (*P* > 0.05). Source data are provided as a Source Data file.

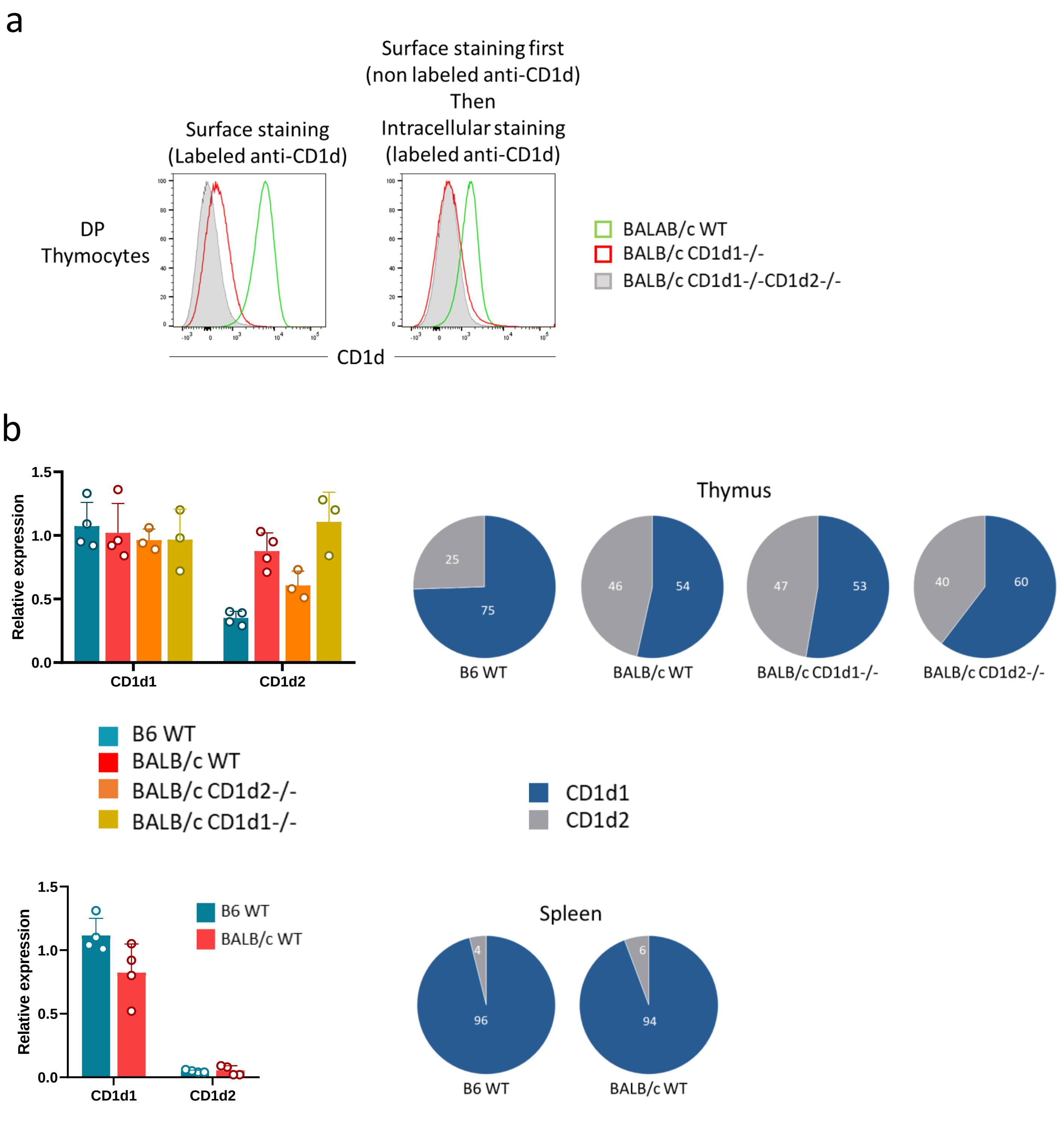

Supplementary Figure 5: **CD1d molecule and CD1D1/CD1D2-encoding transcript expression in distinct mouse stains. a**. Representative surface staining (left) and intracellular staining (right) of CD1d on cortical thymocytes (DP) from BALB/c WT (n= 3), BALB/c CD1d1-/- (n=3), and BALB/c CD1d1-/-CD1d2-/- (n=3) mice. Intracellular staining (right) was performed after blocking the surface molecule with unlabeled anti-CD1d. **b**. Relative expression (Histograms) and Ratio (Pie charts) of CD1D1/CD1D2-encoding transcripts in the thymus from B6 WT (n=4), BALB/c WT (n=4), BALB/c CD1d1-/- (n=3), and BALB/c CD1d2-/- (n=3) and spleen from B6 WT (n=4) and BALB/c WT (n=4) mice. Data are representative of three experiments in a., and four experiments in b., with 7-8-week-old mice. Source data are provided as a Source Data file.

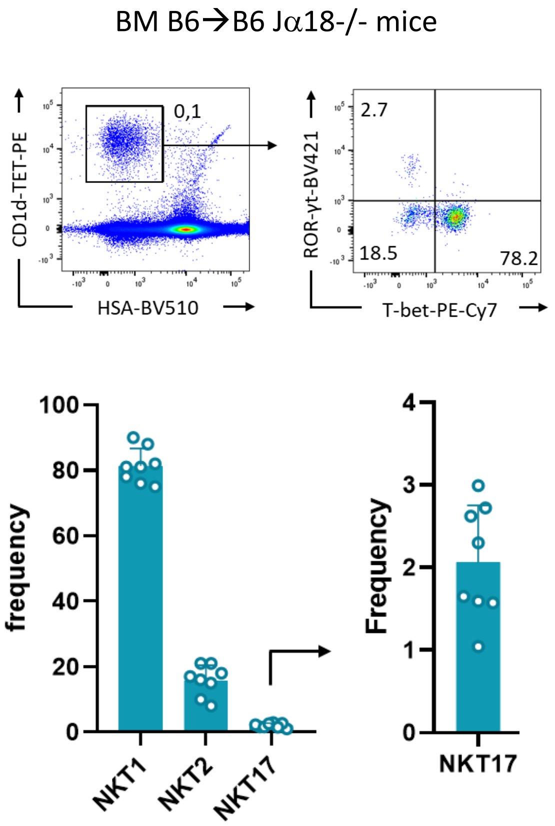

Supplementary Figure 6: Normal NKT cell subset distribution in B6 into B6 Jα18-/- bone marrow chimeras. Representative staining of NKT1 (T-bet^{+}), NKT2 (T-bet^{-}RORγt^{-}), and NKT17 (RORγt^{+}) subsets among thymic NKT cells from B6 into B6 Jα18-/- bone marrow chimeras (n=8). Numbers on dot plots represent frequencies. Individual/mean + SEM of mean frequency for each thymic and splenic NKT cell subset are shown below. Data are representative of three experiments with 7-8-week-old mice. Source data are provided as a Source Data file.

Supplemental Table 1: Lists of Antibodies used in the study

Antibody name	Fluorochrome	Clone	Supplier	references	Dilution
1/ Anti-CD24	BV510	M1/69	Biolegend	101831	(1:200)
2/ Anti-B220	BV510	RA3-6B2	Biolegend	103248	(1:200)
3/ Anti-CD4	BV711	RM4-5	Biolegend	100549	(1:200)
4/ Anti-CD8	BV605	53-6.7	Biolegend	100744	(1:200)
5/ Anti-RORγ	BV421 BD	Q31-378	BD	562894	(1:200)
6/ Anti-Tbet	PECy7	4B10	Biolegend	644824	(1:200)
7/ Anti-CD44	BUV737	IM7	BD	612799	(1:200)
8/ Anti-CD1d	PE	1B1	Invitrogen	12-0011-82	(1:200)
9/ Anti-CD5	BV711	53-7.3	Biolegend	100639	(1:200)
10/ Anti-Ly108	PE	330-AJ	Sony	1273030	(1:200)
11/ Anti-TCRβ	FITC	H57-597	Biolegend	109206	(1:200)
12/ Anti-PLZF	APC	9E12	Invitrogen	17-9322-80	(1:200)
13/ Anti- EGR2	APC	erongr2	Invitrogen	17-6691-82	(1:200)
14/Anti-Eomes	AF647	W17001A	Biolegend	157703	(1:200)
15/ Anti-Vb8.1/8.2	FITC	KJ16-133	eBiosciense:	11-5813-82	(1:100)
16/ Anti-Vβ7	FITC	TR310	Biolegend	118305	(1:100)
17/ Anti-IFN-γ	FITC	XMG1.2	Biolegend	505806	(1:500)
18/ Anti-IL17A	AF647	TC11-18H10	BD	560184	(1:200)
19/ Anti-IL4	BV711	11B11	Biolegend	504133	(1:200)
20/ Anti-CD138	PECy7	281-2	Biolegend	142514	(1:200)
21/ Anti-H-2Kd	AF488	SF1-1.1	Biolegend	116610	(1:500)
22/Anti-H-2Kb	PercP-Cy5.5	AF6-88.5	Biolegend	116516	(1:200)
23/ Anti-NRP2	AF647	C9	Santa Cruz	sc-13117	(1:80)
